# Antimicrobial Hydrogels Based on Cationic Curdlan Derivatives for Biomedical Applications

**DOI:** 10.3390/gels10070424

**Published:** 2024-06-27

**Authors:** Dana M. Suflet, Irina Popescu, Magdalena-Cristina Stanciu, Cristina Mihaela Rimbu

**Affiliations:** 1 Petru Poni Institute of Macromolecular Chemistry, Aleea Grigore Ghica Voda 41A, 700487 Iasi, Romania; ipopescu@icmpp.ro (I.P.); cstanciu@icmpp.ro (M.-C.S.); 2 Faculty of Veterinary Medicine, “Ion Ionescu de la Brad” University of Life Sciences, Mihail Sadoveanu Alley 8, 707027 Iasi, Romania; crimbu@yahoo.com

**Keywords:** curdlan, cationic curdlan, hydrogels, drug delivery system

## Abstract

Hydrogels based on biocompatible polysaccharides with biological activity that can slowly release an active principle at the wound site represent promising alternatives to traditional wound dressing materials. In this respect, new hydrogels based on curdlan derivative with 2-hydroxypropyl dimethyl octyl ammonium groups (QCurd) and native curdlan (Curd) were obtained at room temperature by covalent cross-linking using a diepoxy cross-linking agent. The chemical structure of the QCurd/Curd hydrogels was investigated by Fourier transform infrared spectroscopy (FTIR) spectroscopy. Scanning electron microscopy (SEM) revealed well-defined regulated pores with an average diameter between 50 and 75 μm, and hydrophobic micro-domains of about 5 μm on the pore walls. The high swelling rate (21–24 g_water_/g_hydrogel_) and low elastic modulus values (7–14 kPa) make them ideal for medical applications as wound dressings. To evaluate the possible use of the curdlan-based hydrogels as active dressings, the loading capacity and release kinetics of diclofenac, taken as a model drug, were studied under simulated physiological skin conditions. Several mathematical models have been applied to evaluate drug transport processes and to calculate the diffusion coefficients. The prepared QCurd/Curd hydrogels were found to have good antibacterial properties, showing a bacteriostatic effect after 48 h against *S. aureus*, *MRSA*, *E. coli*, and *P. aeruginosa*. The retarded drug delivery and antimicrobial properties of the new hydrogels support our hypothesis that they are candidates for the manufacture of wound dressings.

## 1. Introduction

The skin is an essential part of the human body, and when cutaneous damage occurs, a wound healing process starts progressing through four physiological stages: haemostasis, inflammation, proliferation, and remodelling [1]. Due to the complexity of the healing mechanisms and the wide variety of wound types, the development of new materials used as wound dressings is still required. The ideal wound dressing should promote rapid healing, with minimal pain for the patient. In particular, it should be non-toxic, biodegradable and biocompatible, have mechanical stability, be able to remove excessive exudate, improve autolytic debridement, reduce inflammation, protect against microbial agents, and maintain adequate moisture for healing [2,3]. Moreover, wound dressings can be classified as follows: a) passive materials (ordinary dressings) that act as a common cover on a wound so that the wound can rehabilitate underneath; and b) interactive materials containing polymeric hydrogels, nanofibers membranes, films and/or foams, which are loaded with drug and permeable to water vapours and atmospheric oxygen [4,5]. Among them, hydrogels are the most promising approach to wound healing because they can provide a suitable environment for tissue regeneration by maintaining a moist environment at the wound site, removing exudate and preventing infection by antimicrobial coating and delivering a therapeutic agent [6,7]. Hydrogel-based dressings can be manufactured from hydrophilic polymers, such as polysaccharides. These have many advantages such as the ability to imitate the tissue, biocompatibility, biodegradability, non-toxicity, and weak inflammatory reactions [8].

The research in the field of biomaterials (dressings) has shown a recent interest in the use of amphiphilic ionic polysaccharides because they combine the hydrophilic properties used in wound dressings with the ability to form micro-domains by self-aggregation in aqueous solutions and to encapsulate drugs in these micro-assemblies [9]. In this respect, the synthesis of polysaccharide derivatives with cationic charges (quaternary ammonium derivatives) has been reported in the literature. So, the quarterisation reaction with lipophilic reagents was applied to different polysaccharides, such as cellulose [10,11], chitosan [12,13], dextran [14], pullulan [15], curdlan [16,17,18], gellan gum [19], and citrus pectin [20] for obtaining the hydrophobic domains and to confer/improve the antibacterial properties.

Curd is a neutral polysaccharide produced by microorganisms, with a linear structure composed of D-glucose units linked by β-(1→3) glucosidic bonds. It is insoluble in water and most organic solvents but dissolves in dimethylsulphoxide, formic acid, some aprotic solvents, and in diluted bases (above 0.2 M NaOH) [21,22]. Curd is currently used in several applications such as the cosmetic industry for skin care [23], biomedical and pharmaceutical industries [24,25] due to its immunological [26,27], anti-inflammatory [28], and non-toxic effects, as well as in the food industry as a food supplement [29]. However, curdlan applications are still limited by its insolubility in water. Thus, the synthesis of soluble derivatives of curdlan with ionic groups such as the sulphate [30], phosphate [31], carboxymethyl [32], and ammonium groups [16,17,33,34] has been reported to resolve this disadvantage. The amphiphilic curdlan derivatives with quaternary ammonium groups and hydrophobic moieties (QCurd) are proven to have antimicrobial properties [17]. In addition, the hydrophobic and electrostatic interactions that can take place between QCurd and anionic hydrophobic drugs can be an advantage in the preparation of active wound dressings.

Considering these aspects, this study reports original results on the obtaining of new QCurd-based hydrogels by covalent cross-linking technique. The morphology of the ionic hydrogels was analysed by SEM, while FTIR spectroscopy was used to confirm the chemical structure. The properties of these new materials that are important for their applications as dressings, such as porosity, kinetics/mechanism of swelling, as well as mechanical properties, were determined. To test their applications as active wound dressings, the hydrogels were loaded with a model drug, diclofenac (DCF), known for its analgesic and anti-inflammatory properties when topically administered. To facilitate future hydrogel development and to understand pharmaceutical dosage forms, the release profiles and transport mechanisms of DCF were studied under simulated physiological conditions. In addition, the antimicrobial and antifungal activities of the new hydrogels were assessed.

## 2. Results and Discussion

### 2.1. Synthesis of QCurd/Curd Hydrogels

**Figure 1:**
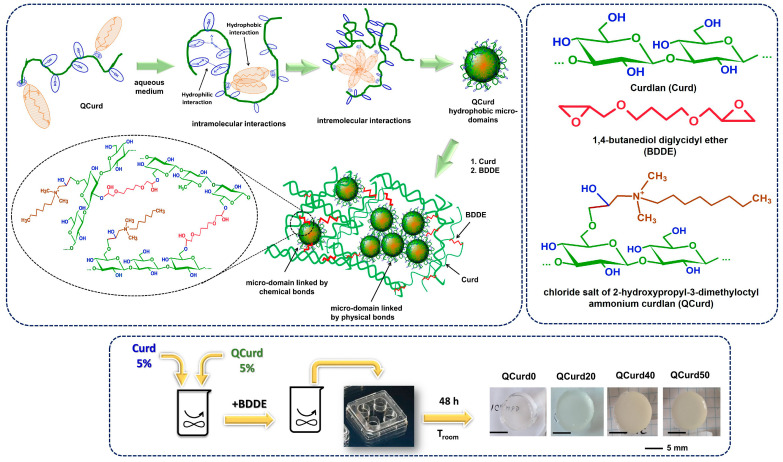
Schematic representation of QCurd/Curd hydrogel formation and optical photomicrographs.

The new hydrogels based on QCurd/Curd were successfully obtained by covalent cross-linking of a mixture of Curd and QCurd with octyl groups using 1,4-butanediol diglycidyl ether (BDDGE) as crosslinker under alkaline conditions. The notation of the hydrogel samples was *QCurdX*, where X represents the mass percent (%) of QCurd from the QCurd/Curd mixture (Table 1). Figure 1 illustrates the chemical structure of the hydrogel components and provides a schematic representation of the arrangement of the polysaccharide chains in its chemical structure. As can be seen from Figure 1, the covalent cross-linking of curdlan resulted in white and transparent hydrogels. (QCurd0), while the presence of QCurd, even in small amounts, led to opaque hydrogels (QCurd20). This aspect could be attributed to the hydrophobic domains within the QCurd structure, which form the “hydrophobic micro-domains” during the dissolution process due to the concentration of the working solution (C_QCurd_ = 5 g/dL), which was above the entanglement concentration (the limit between semi-dilute unentangled and semi-dilute entangled ranges) established to be C_e_ = 1.14 g/dL for this curdlan derivative [17]. Some of these hydrophobic micro-domains were covalently linked through BDDE in the hydrogel structure, while others remained linked by physical interactions such as hydrogen bonds or hydrophobic forces (Figure 1). Table 1 lists the composition and the main characteristics of the prepared hydrogels.

Gel fraction (*GF*, %) was used to quantify the efficiency of the cross-linking. Analysis of the *GF* values (Table 1) revealed a decrease in the degree of cross-linking with increasing QCurd content. The highest *GF* value of 54.0% (g/g) was obtained for the curdlan-based hydrogels (QCurd0). A comparable *GF* value of 50.7% (g/g) was determined for the sample with the lowest QCurd content (20/80, g/g, QCurd20). However, increasing the amount of QCurd in the reaction mixture decreased *GF* values by 42.0% and 31.2% (g/g) for QCurd40 and QCurd 50, respectively. Amphiphilic polymers have been reported in the literature to have the ability to form micelles, micro-domains, or vesicular aggregates in aqueous media [9]. These micro-domains can be bound in the hydrogel structure by covalent bonding (via BDDE) or hydrogen bonds. During the purification process, the hydrogen-bonded micro-domains are typically removed. Additionally, some of the –OH groups at the C6 position of glucosidic rings (the most reactive sites) of the curdlan derivative (DS = 0.4) may not be available to be covalently cross-linked by BDDE. At the same time, the bulky groups of 2-hydroxypropyl dimethyl octyl ammonium can hinder the access of the crosslinking agent to the polysaccharide network. All these aspects led to a decrease in the *GF* values of the hydrogel with the augmentation of QCurd content.

Increasing the percent of QCurd above 50% resulted in the formation of hydrogels, but during the purification process, due to the high degree of swelling, they became difficult to handle.

### 2.2. Characterisation of QCurd/Curd Hydrogels

#### 2.2.1. FTIR Spectra

**Figure 2:**
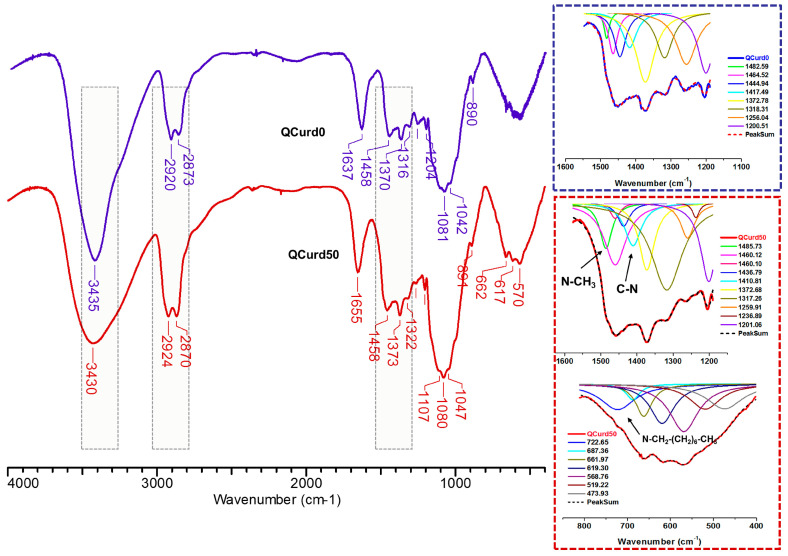
FTIR spectra of the QCurd/Curd hydrogels, without (QCurd0) and with (QCurd50) quaternary derivative.

FTIR spectra of hydrogels without QCurd (QCurd0) and with quaternary derivative (QCurd50) are shown in Figure 2. The spectra of QCurd0 and QCurd50 show the characteristic bands for the parent polymers (Figure S2 from the Supplementary Materials) and a change in the intensity of some bands due to the chemical bonds with BDDE. Thus, the large band at 3435 cm^−1^ (QCurd0) and 3430 cm^−1^ (QCurd50) was attributed to O–H bond stretching vibration, and to the intra- and inter-molecular hydrogen bonds, while the bands from 2920–2924 cm^−1^ and 2873–2870 cm^−1^ were attributed to symmetric and asymmetric stretching vibrations of C–H bonds from CH_2_, CH groups, and CH_2_–O from ether linkages of QCurd50. The increase in the intensity of the band at 2870 cm^−1^ (QCurd50) was associated with CH_3_/CH_2_ groups from quaternary ammonium pendant groups (CH_3_-N, –CH_2_–N). In addition, the widening of the band from 2924 to 2870 cm^−1^ in the case of hydrogels compared with the parent polysaccharides (Figure S2) was associated with the increase in the number of CH_2_/CH groups due to the cross-linker agent (BDDE). The band at 1655 cm^−1^ was associated with bound water in the polysaccharide structure; this region can shift depending on the intensity of the interaction of the polymer with water.

The region from 1500 to 1200 cm^−1^ includes deformational vibrations of groups with local symmetry such as CH_2_ and the numerous C–OH deformations encountered in carbohydrates. Due to the overlap of the bands specific to the new hydrogels with the parent polysaccharides, the identification was achieved only by deconvolution of this region. Thus, the new bands at 1485.73 cm^−1^ corresponding to the stretching vibrations of the methyl/methylene groups linked to the quaternary nitrogen and at 1410.81 cm^−1^ attributed to the C–N stretching vibrations of QCurd50 hydrogel were highlighted (Figure 2, detail) and confirm the presence of QCurd in the hydrogel structure. The region from 1200 to 800 cm^−1^ called the “fingerprint” region of polysaccharides was observed in all samples and contains the stretching vibration of C–O and C–C from the pyranose ring backbones. In addition, the band from 893 to 889 cm^−1^ is called the anomeric region and corresponds to the β-configuration of the anomeric carbon [35]. The large band below 800 cm^−1^, known as the “skeletal region”, is related to the vibration of the carbohydrate skeleton. In this region, a new band at 722 cm^−1^ was evidenced and attributed to the octyl substituent from the quaternary ammonium groups (CH_3_–(CH_2_)_6_–CH_2_–N^+^).

#### 2.2.2. Morphology

**Figure 3:**
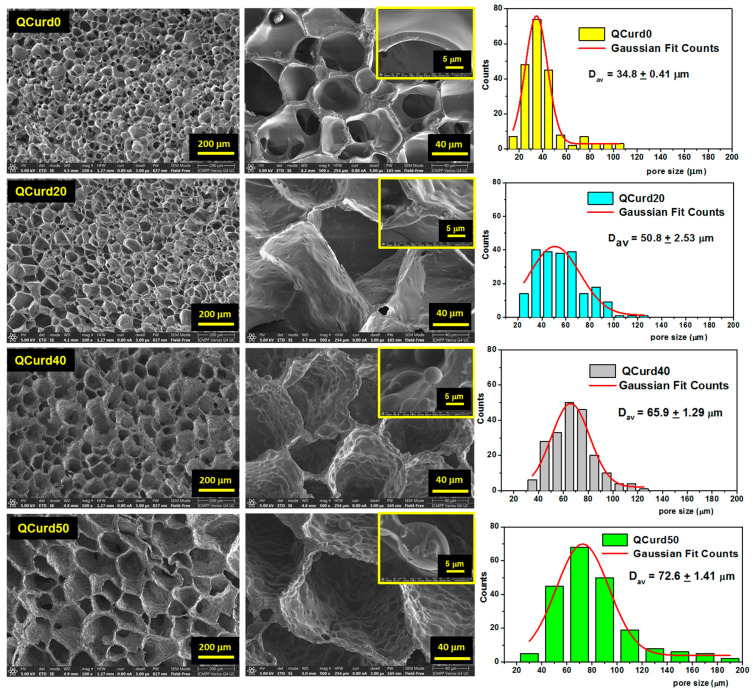
Scanning electron micrographs of QCurd/Curd hydrogels in cross-section and pore size distributions histograms. The image scales are 200 μm, 40 μm, and 5 μm.

The morphological characteristics of hydrogels (i.e., the size and the structure of the pores) can influence their properties such as swelling rate, absorption potential, drug delivery capacity, and mechanical strength. SEM was used to investigate the morphology of QCurd/Curd hydrogels (Figure 3). As can be seen, all hydrogels exhibit a well-defined three-dimensional pore structure, with interconnected pores.

QCurd0 hydrogels display smooth-walled pores of approximately equal size with a narrow size distribution and an average size of 34.8 ± 0.4 μm. The presence of QCurd in low amounts (QCurd20) also generated uniform pores with an average diameter of 50.8 ± 2.3 μm but with slightly wavy walls (Figure 3, insert). SEM micrographics highlight hydrophobic micro-domains covalently crosslinked on the pore walls with the increase in QCurd ratio to 40/60 (g/g) and 50/50 (g/g). The average pore size slightly increases with increasing QCurd content to 65.9 ± 1.3 μm and 72.6 ± 1.4 μm for QCurd40 and QCurd50, respectively. The increase in pore size can be attributed to both the removal of non-crosslinked chains and hydrophobic QCurd micro-domains during the purification process. Furthermore, the SEM micrographs confirm our hypothesis suggesting the arrangement in hydrophobic micro-domains of QCurd derivatives during the dissolution process (Figure 1). The hydrophobic domains exhibit a spherical shape with an average diameter of 11.7 ± 2.7 μm. Similar pore aspects have been reported in the literature for hydrophobic hydrogels [36].

The mass transfer inside the hydrogels and the mechanical properties are influenced to a high extent by their porosity. Therefore, along with determining porosity from SEM micrographs, we also evaluated the porosity of the hydrogels using the solvent immersion method. Thus, an increase in the average porosity was observed from 36% (QCurd0) to 68% (QCurd50) with the increase in the amount of QCurd (Table 1). These results are in good agreement with SEM measurements and data obtained from gel fraction analysis.

Most properties of hydrogels containing ionic polymers, such as swelling capacity, bulking capacity and drug release, depend on the content of cationic groups (exchange capacity). Table 1 shows the exchange capacity values of QCurd/Curd hydrogels determined by conductometric titration (see Supplementary Materials) [17].

#### 2.2.3. Swelling Behaviour

Targeted applications of QCurd/Curd hydrogels as interactive material for wound dressings involve the studies on adsorption capacity, retention kinetics, and transport mechanisms of biological fluids under simulated physiological conditions.

**Figure 4:**
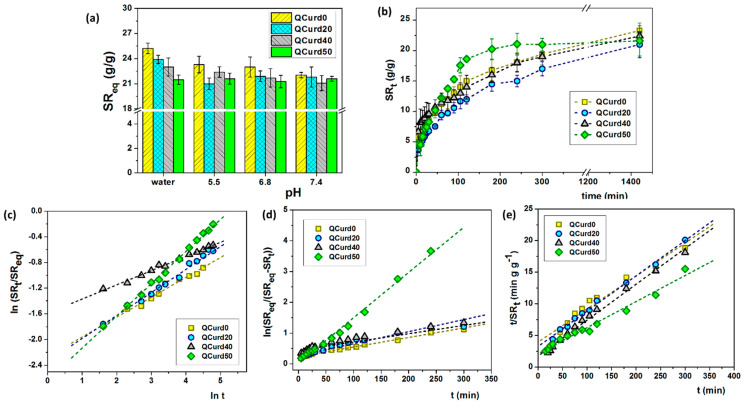
(**a**) Swelling ratio at equilibrium of QCurd/Curd hydrogels in deionised water and various PBS solutions; (**b**) swelling kinetics performed in PBS 5.5 and 32 °C; (**c**) plots of *ln*(*SR_t_*/*SR_eq_*) vs. *ln*(*t*), (**d**) first-order model, and (**e**) second-order model, in PBS 5.5 and 32 °C.

Considering these aspects, the swelling capacity of QCurd/Curd hydrogels was evaluated in water and various PBS solutions (pH 5.5–7.5) at 32 °C (Figure 4a) simulating skin physiological conditions. In our case, the swelling capacity of hydrogels is governed by several forces: (1) electrostatic repulsion forces between positive charges from the quaternary ammonium groups; (2) hydrophobic attractive forces generated by the octyl groups from QCurd; and (3) attractive hydrogen bonds due to the hydrophilic character of polysaccharide chains (Curd and QCurd).

In deionised water, the swelling ratio at equilibrium (*SR_eq_*) gradually decreased with the increase in QCurd content from 25.2 g/g (QCurd0) to 21.5 g/g (QCurd50) due to the hydrophobicity introduced by octyl groups from QCurd. The presence of ions in the swelling medium (PBS with 5.5–7.4 pH) led to shielding of the positive charges from the quaternary ammonium groups, and the swelling process was governed only by the balance between hydrophilic and hydrophobic forces; therefore, the *SR_eq_* values had the same magnitude for all samples (Figure 4a).

The swelling kinetics of the hydrogels play an important role in their applications in the medical field as wound dressings. Swelling kinetics studies showed a gradual swelling of the samples regardless of the medium, reaching equilibrium after 5 h. Figure 4b shows, for example, the swelling kinetics in PBS 5.5 of QCurd/Curd hydrogels. Moreover, the new hydrogels contain quaternary ammonium groups, known to be strong ionic groups, which are found in ionised form throughout the studied pH range, determined that the swelling kinetics was not influenced by the pH of the PBS solution (Figure S3, Supplementary Materials).

The literature reports various studies to determine the mechanism and kinetic parameters of hydrogels swelling [37]. To determine the diffusion mechanism of aqueous solutions into the hydrogel, the swelling data were fitted by the exponential equation:

(1) $$\frac{SR_{t}}{SR_{eq}}=kt^{n}$$

where *SR_t_* and *SR_eq_* are the swelling ratios at the time *t* and at the equilibrium state, respectively, *k* is a characteristic constant of the hydrogel, and *n* is the swelling exponent, which indicates the water transport mechanism. Values of the exponent *n*, calculated from the slope of the plots of *ln*(*SR_t_/SR_eq_*) vs. *ln t*, range between 0.22 and 0.50 (Table 2) indicating a Fickian mechanism of aqueous solutions transport in the hydrogel structure.

In order to establish the mechanism of the swelling processes, for a long time, the experimental data are fitted with two kinetic models. The equation for the *first-order kinetics* model [38,39] can be written as:

(2) $$\frac{dSR_{t}}{dt}=k_{f}\left(SR_{eq}-SR_{t}\right)$$

where *k_f_* (min^–1^) is the rate constant of first-order swelling, and *SR_eq_* and *SR_t_* are the swelling degrees at equilibrium and *t* time, respectively. After integration and applying the initial condition (*SR_t_* = 0 at *t* = 0 and *SR_t_* = *SR_eq_* at *t* = *t_eq_*), the Equation (2) converts to:

(3) $$ln\frac{SR_{eq}}{SR_{eq}-SR_{t}}=k_{f}t$$

The plot of *ln(SR_eq_/(SR_eq_ − SR_t_)* vs. *t* for QCurd/Curd hydrogels is presented in Figure 4d and the *k_f_* parameters are listed in Table 2. Another important parameter for the examination of the swelling kinetics is the coefficient of diffusion (*D*). It can be shown that the *first-order* model is aligned with Fick’s laws of diffusion (Equation (1)). For the cylindrical type of structures, the coefficient *D* can be found by the equation obtained from the arrangement of Fick’s II law and given as [39]:

(4) $$D=\frac{k_{f}r^{2}}{\pi^{2}}$$

In the case of QCurd/Curd hydrogels, a gradual increase in the diffusion coefficient was observed (Table 2). As expected, the highest value of the diffusion coefficient (24.60 × 10^−3^ mm^2^min^−1^) was in the case of sample QCurd50, the sample with a small *GF* and the highest porosity.

The *second-order* kinetics model is usually applied for modelling swelling kinetics when the process is controlled by the hydration of relaxation of the polymeric chains. In this model, the swelling rate depends to a high extent on the sites of the polymer network with swelling capability that have not yet been hydrated by water molecules [39]:

(5) $$\frac{dSR}{dt}=k_{s}{\left(SR_{eq}-SR_{t}\right)}^{2}$$

where *k_s_* is rate constant of swelling in the *second-order* model. After integration and applying the initial condition (*SR_t_* = 0 at *t* = 0 and *SR_t_* = *SR_eq_* at *t* = *t_eq_*) the equation becomes:

(6) $$\frac{t}{SR_{t}}=\frac{1}{k_{s}{\left(SR_{eq}^{t}\right)}^{2}}+\frac{1}{SR_{eq}^{t}}t$$

The swelling kinetic parameters including the rate constant of *second-order* swelling (*k_s_*) and the theoretical degree swelling at equilibrium ($SR_{eq}^{t}$) were determined by fitting the experimental data, *t/SR_t_* vs. *t*, using the second-order equation (Figure 4e). The results are listed in Table 2. In general, the higher *k_s_* value, the faster the swelling process. The calculated values of $SR_{eq}^{t}$ are relatively in agreement with the experimental data, suggesting that the second-order kinetic equation is adequate to describe the swelling of QCurd/Curd hydrogels. Our results are consistent with other reports which describe that the kinetic swelling behaviour of ionic hydrogels follows second-order diffusion kinetics [39,40].

#### 2.2.4. Mechanical Properties

**Figure 5:**
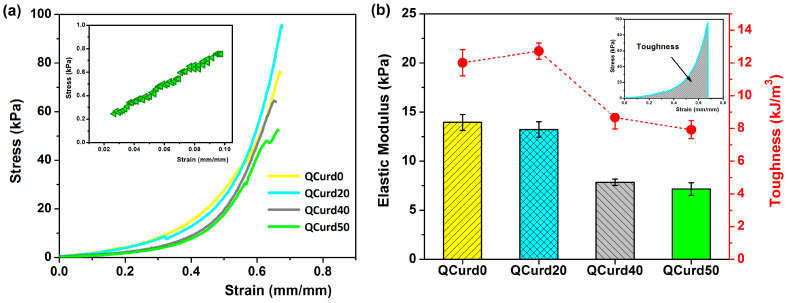
(**a**) Stress–strain profiles of QCurd/Curd hydrogels hydrated in PBS (pH = 5.5); inset figure shows the linear part of the compression curve used to obtain the elastic modulus for QCurd50 sample; (**b**) elastic modulus and toughness values of QCurd/Curd hydrogels.

The mechanical properties of QCurd/Curd hydrogels are important for their applications, especially as wound dressings. The uniaxial compression measurements were performed to evaluate the mechanical behaviour of the hydrogels hydrated in PBS (pH = 5.5). Figure 5 shows the compressive strength curves and the elastic modulus values of QCurd/Curd hydrogels.

The strain–stress curves of the hydrogels swollen in PBS showed profiles characteristic of elastic materials (Figure 5a). The modulus of elasticity, calculated from the slope of the linear part of the compression curves (2–10% strain), decreased insignificantly from 13.9 kPa for QCurd0 to 13.2 kPa for the sample with a 20% quaternary ammonium curdlan derivative. The elastic modulus decreases with increasing QCurd content, reaching values of 7.8 kPa for QCurd40, and 7.2 kPa for QCurd50. This behaviour was due to the sponge structure of the hydrogels with larger pores (see Figure 3). The magnitude of the elastic modulus values is consistent with the literature data on glucan-based hydrogels (6–17 kPa) [41] and close to the elastic modulus of human skin (8.9–9.7 kPa) [42]. Furthermore, although there is a very limited number of studies on the mechanical properties (in the swollen state) of some commercial dressings, elastic moduli of 50–70 kPa have been reported [43,44]. In addition, a decrease in toughness hydrogels from 12 to 12.72 kJ/m^3^ to 7.92 to 8.67 kJ/m^3^ (Figure 5b) was observed for gels with higher QCurd content, probably as a result of the decrease in the degree of cross-linking (*GF*%, Table 1).

#### 2.2.5. Drug Loading and In Vitro Release Studies

Diclofenac sodium (DCF), chosen as a model drug, is the sodium salt form of diclofenac, a non-steroidal drug (NSAID) with analgesic, antipyretic, and anti-inflammatory activity. DCF has both carboxyl groups able to interact through electrostatic forces with the cationic groups of QCurd but also has benzene rings available to interact by hydrophobic forces with the octyl substituents. Thus, the amount of DCF trapped in QCurd0 hydrogel (neutral hydrogel) was reduced (78.01 ± 4 mg/g) due to its retention only by physical forces (hydrogen bonds), while in the case of charged hydrogels containing QCurd the drug loading increases to 175 mg/g (QCurd20), 207 mg/g (QCurd40), and even 241 mg DCF/g for QCurd50 hydrogel (Table 1). This augmentation was due to DCF retention by both electrostatic and hydrophobic forces.

**Figure 6:**
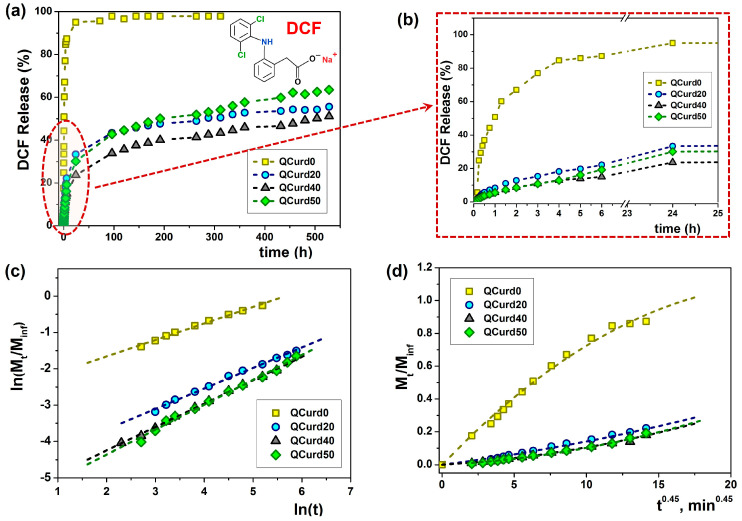
Release dynamics of DCF from loaded QCurd/Curd hydrogels in PBS (pH 5.5) at 32 °C (**a**); detail of the dynamic DCF release and (**b**); plot of Ritger-Peppas (**c**) and Peppas-Sahlin (**d**) models.

Figure 6 shows the release profiles of DCF from hydrogels under simulated physiological conditions (PBS 5.5, at 32 °C). The release rate is influenced by the hydrophobicity/hydrophilicity of the polymer structure, the degree of network swelling in the release medium (in our case, saline pH 5.5) and the electrostatic interactions and hydrogen bonds between the active principle (drug) and the polymer network.

Thus, in the case of the neutral hydrogel (QCurd0), a burst release was observed when 50.8% (mg/g) of the loaded DCF was released in the first hour. The rapid release was caused by the high degree of swelling of QCurd0 and by the diffusion of the drug retained in the structure only by physical forces (hydrogen bonds). As was expected, in the case of ionic hydrogels with different QCurd content, where DCF was retained by hydrophobic, electrostatic, and hydrogen bonds, the release was delayed. Therefore, only 33.4% (QCurd20), 23.6% (QCurd40) and 30.1% (QCurd50) DFC were released after 24 h (Figure 6b). This behaviour was strongly influenced by the occurrence of hydrophobic micro-domains that retain the drug through hydrophobic attractions and electrostatic forces. The swelling rate of the hydrogels was also relatively low (Figure 4b), influencing the drug release. Due to the retarded drug release, even after 3 weeks, less than 60% of the drug was removed from the charged hydrogels containing various amounts of QCurd.

The experimental data were fitted and analysed according to four theoretical diffusion models to elucidate the transport mechanism of DCF from QCurd/Curd hydrogels. Figure 6c,d show the plots of Ritger–Peppas and Peppas–Sahlin models and the release parameters of DCF loaded from hydrogels and the average Akaike Information Criterion (*AIC*, Equation (16)) values for each kinetic model are listed in Table 3.

Using the Ritger–Peppas model Equation (11), the *n* exponent was calculated for all the samples (Figure 6c and Table 3), and the obtained values between 0.45 and 0.69 indicate an anomalous transport mechanism (non-Fickian) for DCF release, meaning that the diffusion and the polymer structure relaxation rates are the same [45]. The Higuchi and zero-order models did not fit the experimental data.

As the *AIC* values show (Table 3), the Peppas–Sahlin (m = 0.45 for cylindrical geometries) and Ritger–Peppas models statistically described the best the experimental data obtained from QCurd/Curd hydrogels. These results indicate that the DCF release mechanism from the QCurd/Curd hydrogels was the result of the contribution both of the diffusion process and the polymer chain relaxation. The studies showed that QCurd content did not influence the drug transport mechanism, which appears to be anomalous regardless of the hydrogel composition.

**Figure 7:**
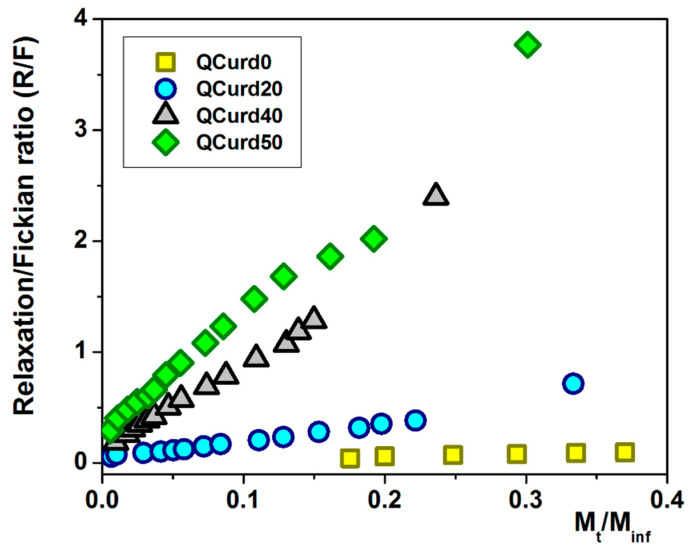
The R/F ratio as a function of the fractural drug release from QCurd/Curd hydrogels.

Using Equations (14) and (15), the ratio between the relaxation contribution (R) and diffusional contribution (F) was calculated during the release of DCF and presented in Figure 7. For the QCurd0 sample, the R/F ratio < 1 confirms that the DCF release was governed by Fickian diffusion, while even small amounts of QCurd in the hydrogel cause the R/F ratio to increase above 1. In this case, the release of the drug was controlled by the relaxation of the polymer chains in the beginning, followed by the diffusion process of DCF.

#### 2.2.6. Antimicrobial Assay

**Figure 8:**
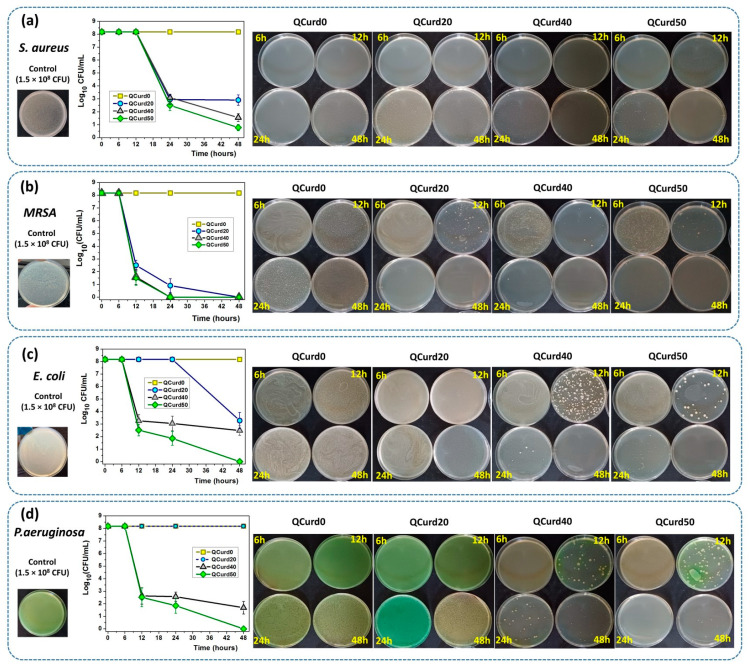
Antimicrobial activity of QCurd/Curd hydrogels against *S. aureus* (**a**), *MRSA* (**b**), *E. coli* (**c**), and *P. aeruginosa* (**d**) after 6, 12, 24, and 48 h of incubation.

The antimicrobial activity of QCurd/Curd hydrogels was tested against two Gram-positive (*S. aureus* and *Methicillin-resistant S. aureus—MRSA*) and two Gram-negative bacterial (*E. coil* and *P. aeruginosa*) species (Figure 8). The remaining colony-forming units (CFU) were measured after incubation of bacterial strains at a density of 1.5 × 10^8^ CFU/mL (Log_10_ CFU/mL from the total viable bacteria = 8.176) in the presence of hydrogels for 6, 12, 24, and 48 h. In principle, the higher the logarithmic reduction values, the more pronounced the antimicrobial effect. It is known that a reduction of 1 Log_10_ corresponds to the killing of 90% of microbes, while 6 Log_10_ destroys 99.9999% of microbial cells.

The results showed that neutral QCurd0 did not show antimicrobial activity, while the ionic hydrogels with various amounts of QCurd in their composition showed different antimicrobial activity, depending on the quantity of QCurd and the type of microorganism. The hydrogel QCurd20 showed, after 48 h of incubation, good antimicrobial activity against Gram-positive bacterial species, which was evaluated from the logarithmic drop of 5.2697 Log_10_CFU/mL (99.9994%) against *S. aureus* (Figure 8a) and a complete antimicrobial activity against *MRSA* (Figure 8b). Lower antimicrobial activity was recorded for Gram-negative species when a logarithmic reduction of 4.8964 Log_10_ CFU/mL (99.9987%) was determined against *E. coli* (Figure 8c) and was not detectable against *P. aeruginosa* (Figure 8d and the Supplementary Materials). As expected, increasing the amount of QCurd in the hydrogel structure resulted in higher antimicrobial activity. Thus, the antimicrobial efficacy of hydrogel QCurd40 was significantly more pronounced against both Gram-positive and Gram-negative species. The results show a logarithmic reduction of 6.6197 Log_10_ CFU/mL (99.9999%) against *S. aureus* and also a complete antimicrobial activity against *MRSA*. The antimicrobial activity against *E. coli* and *P. aeruginosa* was correlated with a logarithmic reduction of 5.6932 Log_10_ CFU/mL (99.9997%) and 6,486 Log_10_ CFU/mL (99.9999%). The best antimicrobial effect against all bacterial species studied, quantified by a result of 7.3979 Log_10_ CFU/mL (99.9999%) against *S. aureus* and an overall effect similar to that of “cid” (100%, infinity), after 48 h, was recorded for the hydrogel with the highest amount of cationic curdlan derivative (QCurd50).

## 3. Conclusions

New QCurd/Curd hydrogels were synthesised by covalent crosslinking of a mixture of curdlan and the curdlan derivative with 2-hydroxypropyl dimethyl octyl ammonium pendant groups. FTIR spectroscopy confirmed the chemical structure of the hydrogels, and SEM revealed well-defined regular pores with 50–72.6 μm size and also confirmed the arrangement in globules of QCurd during the solving process as a result of polymeric self-assembly due to the occurrence of octyl chains. Swelling studies conducted in deionised water and PBS showed a gradual swelling rate at equilibrium, which was reached after 5 h. The values of the exponent *n* between 0.221 and 0.501 indicated a Fickian mechanism of aqueous solutions transport in the hydrogel structure.

Uniaxial compression measurements revealed an elastic behaviour of the hydrogels in the hydrated state, with a decrease in the elastic modulus values from 13.9 kPa to 7.16 kPa with the augmentation of quaternary curdlan derivative content in the hydrogel composition.

New QCurd/Curd hydrogels could be used as active wound dressings. To this aim, the release profile of diclofenac under physiological skin conditions was studied. DCF delivery profiles showed a slow release for all hydrogels containing QCurd. The mathematical models evidenced that the DCF release mechanism appeared to be anomalous (non-Fickian) and governed by the relaxation of the polymer chains in the case of QCurd-based hydrogels, and Fickian diffusion for the Curd-based hydrogels.

The antimicrobial activity studies evidenced an excellent inhibitory effect against bacterial species such as *S. aureus, MRSA*, *E. coli*, and *P. aeruginosa*. Due to their high swelling capacity, elastic properties, gradual release performance, and antibacterial properties QCurd-based hydrogels could be potential wound dressing materials. Future in vivo studies, made for QCurdX, at physiological pH and temperature, can provide more evidence for polymeric effectiveness as materials for dressing wounds.

## 4. Materials and Methods

### 4.1. Materials

Curd (M_w_ ≈ 1000 kDa, determined by Gel Permeation Chromatography) was acquired from Wako Pure Chemical Ind. (Osaka, Japan). The chloride salt of 2-hydroxypropyl-3-dimethyloctyl ammonium curdlan derivative (QCurd) with a degree of substitution of 0.4 was synthesised, in our laboratory, according to the method that was described elsewhere [17]. Epichlorohydrin (ECH), dimethyloctylamine (DMOctA), BDDE, sodium hydroxide (NaOH), and DCF were from Merck-Sigma (Darmstadt, Germany). All reagents were used without further purification and all experiments were performed using deionised water.

### 4.2. Preparation of the QCurd/Curd Hydrogels

The 5% Curd and 5% QCurd solutions were prepared by dissolving them separately in NaOH 1N at room temperature (r.t.). QCurd/Curd hydrogels were prepared by mixing QCurd and Curd solutions in various weight ratios (Table 1). The volume of BDDE was calculated in order to achieve a 1:2 molar ratio between anhydroglucose pyranose units (AGU) and epoxy groups of BDDE (AGU:Epoxy). After the slow addition of the crosslinker, the mixtures were poured into Petri dishes (d = 50 mm) or in culture cell plates with four wells (d = 10 mm) and stored for 48 h at r.t. to allow the chemical crosslinking reaction of the polymeric chains. The hydrogels were purified by washing three times with deionised water and recovered using a laboratory freeze dryer ALPHA 1-2 LD (Martin Christ GmbH, Osterode, Germany).

For the quantitative determination of the quaternary groups in QCurd/Curd hydrogels, conductometric titrations were performed using the protocol described in reference [17]. Briefly, the hydrogel samples were weighed, immersed overnight in 25 mL of water, and then brought to the ionised form with 0.1 N HCl solution. The excess of HCl was measured by titration with 0.1 M NaOH solution using a conductivity meter CMD 210 (Radiometer, Copenhagen, Denmark) equipped with a CDC 865 cell (Figure S1, Supplementary Materials).

### 4.3. QCurd/Curd Hydrogels Characterisation

#### 4.3.1. Structural Characterisation

FTIR spectra of QCurd/Curd hydrogels were recorded on KBr pellet using a Bruker Vertex 70 spectrometer (Bruker, Vienna, Austria).

#### 4.3.2. Gel Fraction

In order to evaluate the ratio between the polymers (QCurd and Curd) involved in the covalent cross-linking reaction from the total amount of the polymers, *GF* was measured. Briefly, the hydrogels were weighed (*W_i_*, g—the initial weight of the dry sample) after obtaining and freeze-drying, and then they were washed with deionized water several times when the non-cross-linked polymer chains and unreacted cross-linking agent were removed. The washed hydrogels were freeze-dried again and weighed (*W_p_*, g—weight of the purified sample in the dried state). The gel fraction (*GF*, %) that can be assimilated with the degree of cross-linking [46], was calculated as:

(7) $$GF\left(\%\right)=W_{p}/W_{i}\times 100$$

#### 4.3.3. Morphology and Porosity Measurement

A Verios G4 UC Scanning Electron Microscope (Thermo Scientific, Brno, Czech Republic) was used to investigate the cross-section morphology of QCurd/Curd hydrogels. The hydrogels were previously coated with platinum using a Leica EM ACE200 Sputter coater. The pore size and size distribution were determined from SEM micrographics using ImageJ 1.48v (Wayne Rasband National Institutes of Health, Bethesda, MD, USA) and OriginPro 8.5.0 (OriginLab Corporation, Northampton, MA, USA) software.

The porosity (*P*, %) of the hydrogels was estimated using the solvent immersion method, with slight modifications [47,48]. Briefly, the freeze-dried QCurd/Curd hydrogels with cylindrical shapes were weighed (*W_d_*, g) and immersed in anhydrous ethanol (99.5%) at r.t. After 15 min, the hydrogels were taken out from the medium, the ethanol excess was removed, and then the samples with ethanol were weighed (*W_s_*, g). Measuring the volume of the sample before immersing (*V*, cm^3^), and knowing the density of anhydrous ethanol at room temperature (*ρ* = 0.785 g/cm^3^), the porosity of the hydrogels could be calculated using the Equation (8):

(8) $$P\;\left(\%\right)=\left(W_{s}-W_{d}\right)\times 100\;/\rho \cdot V$$

The test was repeated 3 times, and the data are expressed as means ± standard deviation.

#### 4.3.4. Swelling Ratio

The capacity of the QCurd/Curd hydrogels to swell was evaluated from the swelling ratio by gravimetric method. The swelling capacity was evaluated both in deionised water and phosphate buffer solutions with pH 5.5, 6.8, and 7.4 (PBS, KH_2_PO_4_ + Na_2_HPO_4_, 0.0667 M) at 32 °C, in order to simulate the physiological conditions. Briefly, the dried and weighted hydrogel (*W_d_*, g) was immersed in the medium, and at selected time intervals, the samples were carefully withdrawn, the aqueous excess was removed with wet filter paper, then the samples were weighed (*W_s_*, g). The swelling ratio (*SR*, g_water_/g_hydrogel_) was calculated according to Equation (9):

(9) $$SR\;\left(\%\right)=(W_{s}-W_{d})/W_{d}$$

#### 4.3.5. Mechanical Properties

Compressive test of the hydrogels was performed using Texture Analyser (Brookfield Texture PRO CT3^®^, Brookfield Engineering Laboratories Inc., Middleborough, MA, USA) equipped with a 4500 g load cell and a TA11/1000 probe (25.4 mm diameter), at room temperature.

The hydrogels swelled in PBS with pH 5.5 (cylinder with diameter—17 mm, height—8.5 mm) were tested under uniaxial compression till 70% deformation, at a speed test of 0.2 mm/s. Compression stress and strain values were calculated from the values of force and deformation, knowing the cross-section area and the initial height [46,48]. The elastic modulus (*E*, kPa) was calculated from the linear part of stress–strain curves between 2 and 10% compression. The toughness of the hydrogels, which is the work to fracture, was determined from the area under the compressive stress–strain curve of the sample [49].

#### 4.3.6. Loading Capacity and Diclofenac Release from QCurd/Curd Hydrogels

DCF-loaded hydrogels were prepared by the swelling–diffusion method. Hydrogel (approx. 25 mg) was immersed into 20 mL of DCF aqueous solution (1 mg/mL) and kept for 48 h at r.t. The drug used in all the sorption experiments was in excess, the ratio between the drug and the total exchange capacity of hydrogel QCurd50 was 2:1.

Finally, the loaded hydrogels were washed with distilled water three times and freeze-dried. The amount of DCF incorporated in the hydrogel was determined by the spectrophotometric method, at λ = 275 nm, using a UV–Vis spectrophotometer (Thermo Fisher Scientific Evolution 201, Waltham, MA, USA). The amount of DCF loading (*L_DCF_*) was evaluated by Equation (10), using a previously made calibration curve in water.

(10) $$L_{DCF}\left(\%\right)=\frac{Amount\;of\;drug\;in\;hydrogel}{Amount\;of\;drug\_loaded\;hydrogel}\times 100$$

The in vitro drug release study was carried out in PBS (pH 5.5) at 32 °C (±0.1 °C), as the simulated skin conditions. The DCF-loaded hydrogels (25 mg) were immersed in PBS (30 mL), under stirring (100 rpm). A 3 mL amount of the release medium was removed at different time intervals and replaced with the same volume of fresh buffer. The concentration of DCF in the release medium was determined by UV–Vis spectrophotometry (λ = 275 nm) based on calibration curves previously obtained under the same conditions. The experiment was conducted in triplicate.

#### 4.3.7. Mathematical Analysis of the Drug Transport Mechanism

In order to study the DCF transport mechanism from loaded QCurd/Curd hydrogels, the experimental data were fitted with four diffusion models. *Model 1* was an empirical equation proposed by Peppas et al. considering a time-dependent power law function [50,51]:

(11) $$\frac{M_{t}}{M_{\infty }}=kt^{n}$$

where *M_t_* is the absolute cumulative amount of the DCF released at time *t*, *M_∞_* is the absolute cumulative amount of drug release after infinite time, *k* is a structural/geometric constant for a particular system that is dependent on the polymer characteristics at the elapsed time *t* and *n* is the diffusional exponent characteristic of the release mechanism of the system. For a cylindrical geometry [50], the value of *n* = 0.45 indicated Case I diffusion (Fickian diffusion-controlled drug release) in which the rate of solvent penetration was much smaller than the rate of polymer chain relaxation and the system controlled by diffusion. If the value of *n* is within a range of 0.45 to 0.89, this indicates an anomalous (non-Fickian) diffusion mechanism where the diffusion and relaxation rates had the same magnitude. Sometimes, the solvent diffusion rate was much below the polymer chain relaxation rate, where the *n* value can be observed below 0.45. This situation was also classified as Fickian diffusion, called “Less Fickian” behaviour (pseudo-Fickian diffusion). Value of *n* = 0.89 indicated Case II transport/zero order (swelling-controlled drug release) which describes the diffusion process as much faster than the relaxation process and the system controlled by relaxation. Occasionally, the values of *n* > 0.89 have been observed, describing super Case II transport of drug release mechanism and indicating that the drug was released by both diffusion and relaxation of the polymer chains. *Model 2* represents a zero-order drug release, which is expressed by the following equation:

(12) $$\frac{M_{t}}{M_{\infty }}=k_{0}t$$

where *k*_0_ is the kinetic dissolution constant. *Model 3*, based on the Higuchi equation, is used to describe the Fickian diffusion of a drug from an insoluble matrix [52]:

(13) $$\frac{M_{t}}{M_{\infty }}=k_{H}t^{1/2}$$

where *k_H_* is a release kinetic constant. *Model 4* is based on the Peppas–Sahlin equation with exponent *m* fixed to 0.45 [45], which could also be used to describe the release behaviour of dynamically swelling hydrogels, which accounts for the coupled effects of Fickian diffusion and Case II transport:

(14) $$\frac{M_{t}}{M_{\infty }}=k_{1}t^{m}+k_{2}t^{2m}\;\;\;\overset{m=0.45}{\Rightarrow }\;\frac{M_{t}}{M_{\infty }}=k_{1}t^{0.45}+k_{2}t^{0.9}$$

where the first term of this equation represents the contribution of Fickian diffusion, and the second term refers to the macromolecular relaxation contribution on the overall release mechanism. Using the estimated parameters *k*_1_ and *k*_2_ obtained from fitting Equation (14), the ratio of relaxation (*R*) and the diffusional (*F*) contributions were calculated using Equation (15):

(15) $$\frac{R}{F}=\frac{k_{2}}{k_{1}}t^{0.45}$$

To establish the model most appropriate to describe the drug release mechanism, the Akaike Information Criterion (*AIC*) was used. *AIC* was calculated as [53]:

(16) AIC=N(lnSSR)+2p

where *N* is the number of experimental data, *SSR* is the square residual sum and *p* is the number of parameters in the model.

#### 4.3.8. Antimicrobial Assay

The antimicrobial activity of QCurd/Curd hydrogels was tested against the bacterial species *Staphylococcus aureus* ATCC 25923 (*S. aureus*), *Methicillin-resistant Staphylococcus aureus* (*MRSA*) ATCC 43300, *Escherichia coli* ATCC 25922 (*E. coli*), and *Pseudomonas aeruginosa* ATCC 9027 (*P. aeruginosa*) using the “time-kill” assay, which was adapted for testing these types of samples [48,54].

Bacterial suspensions with a bacterial density of 1.5 × 10^8^ CFU/mL (corresponding to a standard turbidity of 0.5 McFarland) were prepared from the 24 h bacterial cultures. Each hydrogel sample was added to 5 mL of the bacterial suspension, shaken by vortexing (1 min) and at each predetermined time point (6, 12, 24, and 48 h) 1 mL of the suspension was removed and distributed into sterile Petri plate (Ø 90 mm), adding the Mueller–Hinton Agar (Bio-Rad Laboratories) culture medium, melted and cooled to 45 °C. After homogenisation and solidification of the medium, the plates were incubated for 24 h at 37 °C in a microbiological thermostat (Binder BD23, Roth, Germany). The antimicrobial efficacy was evaluated by calculating the logarithmic reduction (LR) and the percentage on the logarithmic scale (LR%) of the values resulting from the comparison of the number of bacterial colonies (colony forming units, CFU/mL) from the initial suspension (control sample) with the colony forming units still alive after contact with (see Supplementary Materials). Experiments were carried out in triplicate.

## Figures and Tables

**Table 1:** Chemical composition and the main characteristics of the QCurd/Curd hydrogels.

Sample	QCurd/Curd (g/g)	[AGU:epoxy] (molar ratio)	*EC* (meq./mL)	*GF* (%, g/g)	*P* (%, g/g)	*L_DCF_*, (mg/g)
QCurd0	0/100	1:2	-	54.0 (±1.65)	36.2(±0.58)	78.0(±4)
QCurd20	20/80		0.13 (±0.15)	50.7 (±1.02)	59.6(±1.9)	175.1(±10)
QCurd40	40/60		0.30 (±0.20)	42.0 (±0.21)	64.4(±2.5)	207.9(±13)
QCurd50	50/50		0.40 (±0.26)	31.2 (±0.86)	61.3(±2.0)	241.5(±12)

**Table 2:** Swelling kinetics parameters of QCurd/Curd hydrogels in PBS 5.5, 32 °C.

Sample	Swelling Exponent		First-Order		Second-Order Linear Regression	
	*n*	*k*	*k_f_* × 10^3^	*D* × 10^3^ (mm^2^·min^−1^)	*k_s_* × 10^4^	$SR_{eq}^{t}$ (g_water_/g_hydrogel_)
QCurd0	0.309	0.103	3.1	1.83	6.89	19.16
QCurd20	0.358	0.096	4.2	8.47	9.54	17.92
QCurd40	0.221	0.204	2.7	5.50	18.40	17.64
QCurd50	0.501	0.116	14.7	24.60	8.05	24.27

**Table 3:** Release parameters of DCF from the loaded QCurd hydrogels together with the *AIC* values for different kinetic models.

Sample	Ritger–Peppas			Zero-Order		Higuchi– Fickian		Peppas–Sahlin			Optimum Model
	*k* × 10^2^	*n*	*AIC*	*k*_0_ × 10^2^	*AIC*	*k_H_* × 10^2^	*AIC*	*k*_1_ × 10^2^	*k*_2_ × 10^3^	*AIC*	
QCurd0	49.17	0.45	−52.02	81.17	−20.21	46.38	−35.58	9.15	1.90	−59.61	Peppas–Sahlin
QCurd20	8.31	0.51	−91.78	10.74	−50.13	8.75	−61.13	1.64	0.20	−49.28	Ritger–Peppas
QCurd40	5.51	0.64	−110.13	7.69	−25.90	6.11	−80.95	0.55	0.50	−110.08	Peppas–Sahlin
QCurd50	5.37	0.69	−96.93	7.41	−71.51	6.03	−62.05	0.42	0.60	−105.12	Peppas–Sahlin

## Data Availability

The data presented in this study are available on request from the corresponding author.

## Supplementary Materials

The following supporting information can be downloaded at: https://www.mdpi.com/article/10.3390/gels10070424/s1, Figure S1: Conductometric titration curve of QCurd/Curd hydrogel; Figure S2. FTIR spectra of the Curd and QCurd; Figure S3. Swelling kinetics of QCurd/Curd hydrogels performed in PBS 7.4 (a) and 6.8 (b), at 32 °C; Table S1: The results of the antimicrobial activity of QCurd/Curd hydrogels.
