# Supplementary material for: Antimicrobial Hydrogels Based on Cationic Curdlan Derivatives for Biomedical Applications
Source: Gels. 2024 Jun 27;10(7):424. doi: 10.3390/gels10070424 (PMC11276469; doi:10.3390/gels10070424)
Supplement: Supplementary file 1 [file gels-10-00424-s001.zip › gels-3057264-supplementary.pdf]

Supplementary Information (SI)

Exchange Capacity of QCurd/Curd hydrogels

The exchange capacity of the hydrogels (EC) was determined by titration method, using a 712 Metrohm Conductometer (Herisau, Switzerland) with 6.0908.110 cell for the conductometric titrations. Briefly, 100 mg hydrogels were swollen in 20 mL twice-distilled water for 6 h. After that, an excess of 0.1N HCl solution (1 mL) was added and maintained for 2 h under gentle stirring, and then the solution was subjected to conductometric titration with a 0.1N NaOH solution. The exchange capacity (EC, meqiv./g) was calculated from conductometric data and expressed as meq. ammonium groups / g dried hydrogel.

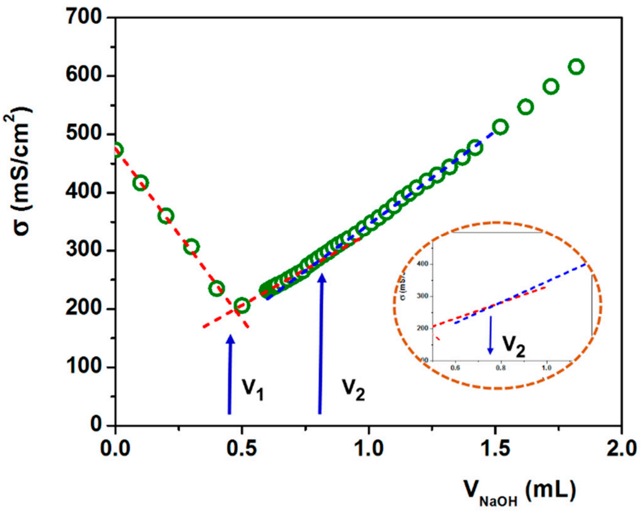

Figure S1. Conductometric titration curve of QCurd/Curd hydrogel

$$EC = \frac{(V_2 - V_1)N_{NaOH}10^{-3}}{m} \quad \text{(S1)}$$

m – mass of sample (g) taken to titration, N_{NaOH} – the normal concentration of NaOH solution; V_1 and V_2 – the volume (mL) of NaOH used for the titration of 1 mL 0.1N HCl added in excess and for the ammonium groups from the hydrogel, respectively

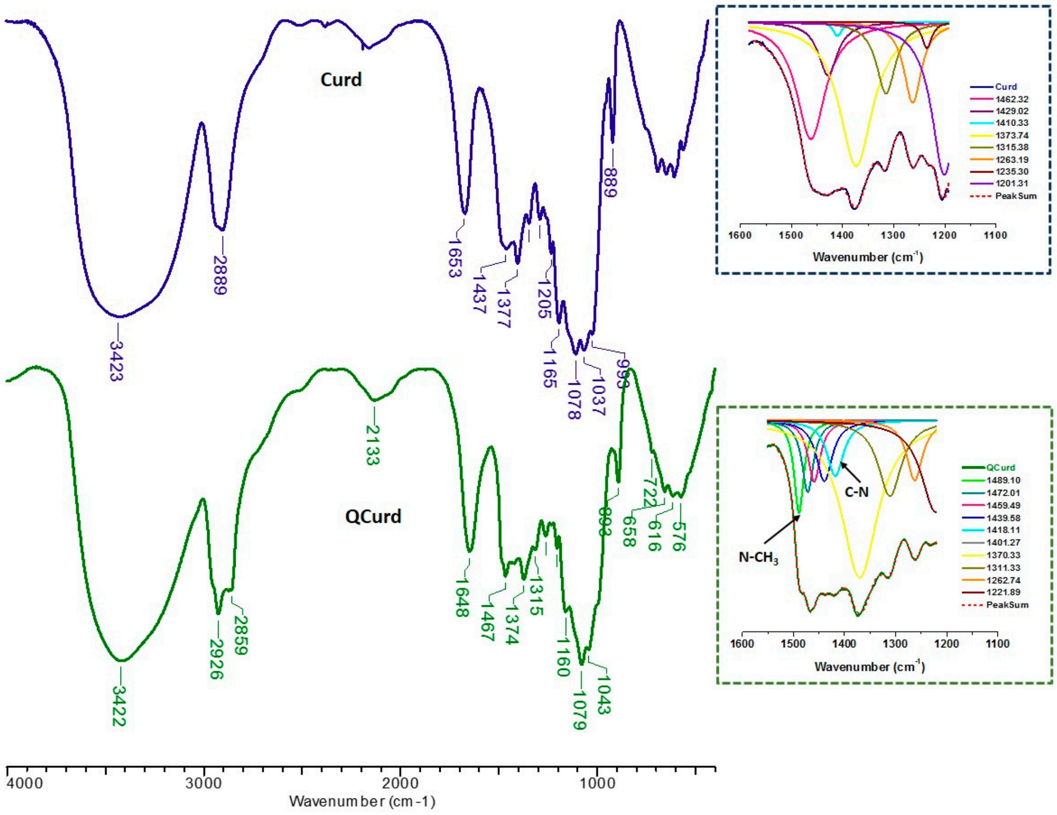

Figure S2. FTIR spectra of the Curd and QCurd

Swelling behaviour

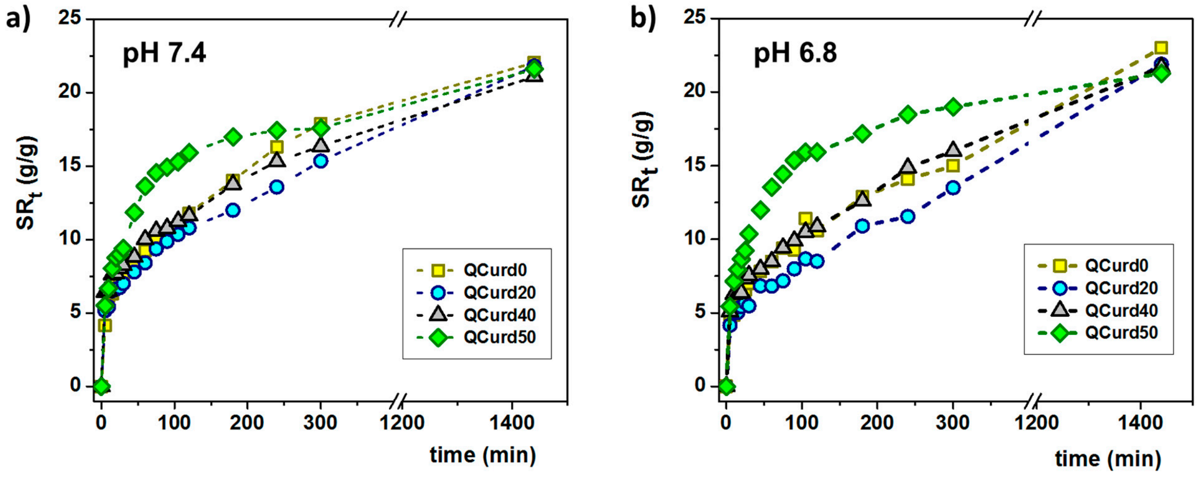

Figure S3. Swelling kinetics of QCurd/Curd hydrogels performed in PBS 7.4 (a) and 6.8 (b), at 32ºC

Antimicrobial assay

The antimicrobial efficacy is evaluated by calculating the logarithmic reduction (*LR*) and the percentage on the logarithmic scale (*LR*%) of the values resulting from the comparison of the number of bacterial colonies (colony forming units, CFU/ml) from the initial suspension (control sample) with the number of microorganisms still alive after contact with the test samples.

$$\text{Log}_{10}\text{ reduction } (LR) = \text{mean } \log_{10}\text{ (microbial population)} \times \text{mean } \log_{10}\text{ (surviving population)} \quad \text{(S2)}$$

$$\textit{Percent Reduction } (\%) = 100 \times (1 - 10^{-LR}) \quad \text{(S3)}$$

To facilitate the evaluation of the results, the CFU/mL values were converted to $\log_{10}$ values and expressed as a percentage, where a reduction of 1 Log_{10} is known to correspond to a bacterial reduction of 90%, while a reduction of 6 Log_{10} corresponds to a reduction of 99.9999%.

Table S1. The results of the antimicrobial activity of QCurd/Curd hydrogels which were obtained using the `kill time` technique and evaluated at different contact times by quantifying the logarithmic reduction values

Bacteria	Times	Samples			
		QCurd0	QCurd20	QCurd40	QCurd50
S. aureus *ATCC 25923*	**Control**	1,5x10^{8}CFU/mL	1,5x10^{8}CFU/mL	1,5x10^{8}CFU/mL	1,5x10^{8}CFU/mL
	**Log$_{10}$**	8.17609	8.17609	8.17609	8.17609
	**6h**	UQ	UQ	UQ	UQ
	**12 h**	UQ	UQ	UQ	UQ
	**24 h**	UQ	892	1267	316
	**48 h**	UQ	806	36	6
	**Log reduction (48 h)**	0	5.2697	6.6197	7.3979
	**% reduction (48 h)**	0	99.9994	99.9999	99.9999
MRSA ATCC 43300	**Control**	1,5x10^8	1,5x10^8	1,5x10^8	1,5x10^8
	**6h**	UQ	UQ	UQ	UQ
	**12 h**	UQ	316	30	38
	**24 h**	UQ	8	0	0
	**48 h**	UQ	0	0	0
	**Log reduction (48 h)**	0	Infinity	Infinity	Infinity
	**% reduction (48 h)**	0	100	100	100
E. coli *ATCC 25922*	**Control**	1,5x10^8	1,5x10^8	1,5x10^8	1,5x10^8
	**6h**	UQ	UQ	UQ	UQ
	**12 h**	UQ	UQ	1839	326
	**24 h**	UQ	UQ	1121	71
	**48 h**	UQ	1904	304	0

	**Log reduction (48 h)**	0	4.8964	5.6932	Infinity
	**% reduction (48 h)**	0	99.9987	99.9997	100
P. aeruginosa ATCC 9027	**Control**	1,5x10^{8}	1,5x10^{8}	1,5x10^{8}	1,5x10^{8}
	**6h**	UQ	UQ	UQ	UQ
	**12 h**	UQ	UQ	430	326
	**24 h**	UQ	UQ	368	71
	**48 h**	UQ	UQ	49	0
	**Log reduction (48 h)**	0	0	6,486	Infinity
	**% reduction (48 h)**	0	0	99.9999	100

CFU = colony-forming unit; UQ = unquantifiable; h= hours; Control - 0,5 McFarland turbidity
